# Bibliometric analysis of electroencephalogram research in mild cognitive impairment from 2005 to 2022

**DOI:** 10.3389/fnins.2023.1128851

**Published:** 2023-03-20

**Authors:** Mingrui Liu, Baohu Liu, Zelin Ye, Dongyu Wu

**Affiliations:** ^1^Department of Rehabilitation, Wangjing Hospital, China Academy of Chinese Medical Sciences, Beijing, China; ^2^Department of Cardiovascular, Guang’anmen Hospital, China Academy of Chinese Medical Sciences, Beijing, China

**Keywords:** mild cognitive impairment, electroencephalogram, CiteSpace, bibliometrics, Alzheimer’s disease

## Abstract

**Background:**

Electroencephalogram (EEG), one of the most commonly used non-invasive neurophysiological examination techniques, advanced rapidly between 2005 and 2022, particularly when it was used for the diagnosis and prognosis of mild cognitive impairment (MCI). This study used a bibliometric approach to synthesize the knowledge structure and cutting-edge hotspots of EEG application in the MCI.

**Methods:**

Related publications in the Web of Science Core Collection (WosCC) were retrieved from inception to 30 September 2022. CiteSpace, VOSviewer, and HistCite software were employed to perform bibliographic and visualization analyses.

**Results:**

Between 2005 and 2022, 2,905 studies related to the application of EEG in MCI were investigated. The United States had the highest number of publications and was at the top of the list of international collaborations. In terms of total number of articles, IRCCS San Raffaele Pisana ranked first among institutions. The Clinical Neurophysiology published the greatest number of articles. The author with the highest citations was Babiloni C. In descending order of frequency, keywords with the highest frequency were “EEG,” “mild cognitive impairment,” and “Alzheimer’s disease”.

**Conclusion:**

The application of EEG in MCI was investigated using bibliographic analysis. The research emphasis has shifted from examining local brain lesions with EEG to neural network mechanisms. The paradigm of big data and intelligent analysis is becoming more relevant in EEG analytical methods. The use of EEG to link MCI to other related neurological disorders, and to evaluate new targets for diagnosis and treatment, has become a new research trend. The above-mentioned findings have implications in the future research on the application of EEG in MCI.

## Introduction

Mild cognitive impairment (MCI) is a clinical syndrome characterized by aging in cases who have short-term or long-term memory loss, while they have no significant daily functional handicap. MCI patients have a high rate of conversion to dementia, particularly Alzheimer’s disease (AD) (Chertkow, 2002). Furthermore, it has been identified as a premonitory stage of several neurodegenerative dementias, including Lewy body dementia, Parkinson’s disease dementia, and frontotemporal dementia (de Mendonça et al., 2004; Tröster, 2008; Aarsland, 2016). The probability that MCI may advance to dementia within a year varies from 10 to 54% (Petersen et al., 1999; Schmidtke and Hermeneit, 2008), and conversion rate depends on the specific MCI subtype (Ferman et al., 2013). Patients’ global cognitive status is typically assessed through clinical cognitive tests such as the Minimum Mental State Examination (MMSE), which is commonly used to screen for dementia and MCI (Folstein et al., 1975). However, neuropsychological tests are vulnerable to subjectivity and are impacted by factors, such as education, culture, attention, etc., (Chertkow, 2002). There is a need for economic, objectively quantifiable, and non-invasive measures of identification and diagnosis to increase therapeutic options and avoid progression from MCI to dementia. Currently, most EEG exams or training models are based on historical diagnoses using the MMSE (Al-Nuaimi et al., 2021; Trinh et al., 2021), which reinforces the need for improved and more reliable diagnostic tools.

Electroencephalogram (EEG) is a promising measurement. EEG is a method for capturing spontaneous electrical activity produced in the cerebral cortex that uses numerous electrodes placed on the scalp (Paul and Dittrichová, 1977). More than 80 years ago, Hans Berger, who invented the term EEG, was the first to record cortical oscillatory activity in humans from the surface of the skull (Berger, 1929). Because EEG signals are stochastic and multidimensional, a wide range of analytical approaches have been proposed to quantify and discover various features of cortical oscillatory activity and their functional roles (Khanna et al., 2015). Few examples included non-linear analysis of the chaotic nature of reactive neural systems, time-frequency analysis to determine event-related synchronization or event-related desynchronization (Roach and Mathalon, 2008), microstate analysis based on scalp electric field topographic map clustering (Khanna et al., 2015), and so forth.

Bibliometrics is a new approach to research output statistics that may provide qualitative and quantitative characteristics of literature for investigators by analyzing measurement indicators, such as country, journal, institution, and keywords (Muslu, 2018). This approach can be used to develop guidelines, pinpoint research hotpots, and predict research trends (Guler et al., 2016). However, no bibliometric research on EEG for MCI has yet been reported. Thus, the present study aimed to use bibliometric analysis software to trace hotspots in this field from 2005 to 2022 and describe prospects for future research.

## Methods

### Data source

In this study, the Web of Science Core Collection (WosCC) database was selected as the data source, while to ensure comprehensive and accurate retrieval of the data, the Science Citation Index Expanded (SCI-Expanded) and Social Sciences Citation Index (SSCI) were also utilized. The WosCC is a classic citation database, which provides necessary information, including literature abstracts, research collaborations, and citations for bibliometric analysis (Yeung et al., 2020). As a result, the WoSCC is recognized as the primary online database for bibliometric analysis (Zhao et al., 2018).

### Search strategy

**FIGURE 1:**
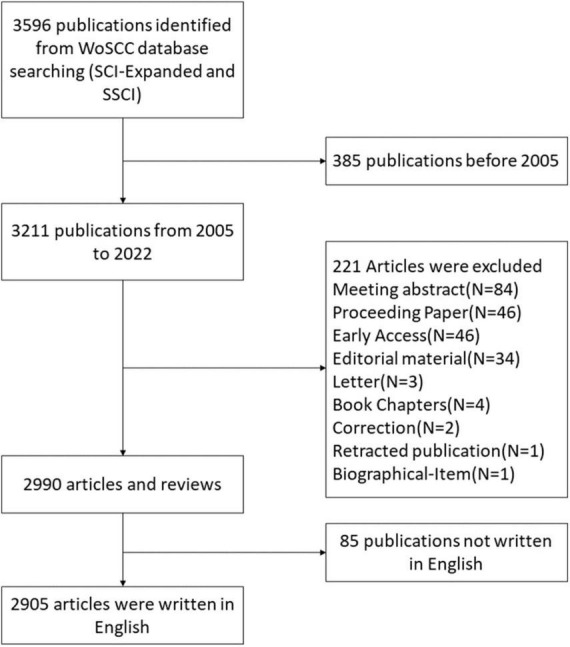
Literature search process.

The data retrieval strategy was summarized as follows: #1: TS = (“Cognitive Dysfunction*” OR “Cognitive impairment*” OR MCI OR “Mild Cognitive Impairment*” OR “Mild Neurocognitive Disorder*” OR “Cognitive decline*”); #2: AND TS = (Electroencephalography OR EEG OR Electroencephalogram*); the ultimate dataset: #1 AND #2. The use of a truncation symbol “*” prevented missed detections and improved retrieval effects. Only English-language studies were included, and duplicate articles were removed. The time of search period was between 1 January 2005 and 30 September 2022. To prevent bias incurred by routine database updates, literature search was conducted on a particular date (20 October 2022). A total of 2,905 studies were retrieved, including reviews and original articles. Figure 1 depicts the search strategy.

### Analytical tools

All data collected were exported for further visual analysis using bibliometric analysis software, such as CiteSpaceV (ver. 6.1.R3), VOSviewer (ver. 1.6.16), and HistCite Pro (ver. 2.1).

CiteSpace, a tool for citation visualization and analysis, concentrates primarily on prospective data found in scientific studies. In the present study, CiteSpace software was used to perform dual-map overlay and clustering of co-citation references. The clustering results were clear and stable when the modularity Q was greater than 0.3 and the mean silhouette value was higher than 0.5 in the cluster analysis. The centrality of a node is an indicator that shows its importance in a network, and the higher the centrality of a node, the greater its influence (Chen, 2006).

In contrast to generally used bibliometric programs, VOSviewer concentrates on graphical descriptions of bibliometrics (van Eck and Waltman, 2010). Nodes on the VOS viewer map are corresponded to distinct parameters, such as countries, institutions, or keywords. Weighting attributes, including the number of publications or quantity of citations is used to determine node size. The colors of nodes and lines represent different clusters. To evaluate the strength of the connections, the total link strength (TLS) index was used, representing the overall co-authorship and co-citation link strength among countries, institutions, or keywords (Pei et al., 2022).

HistCite is a comprehensive citation analysis software that may be used to trace the evolution of a research topic and analyze and visualize direct citation linkages between scientific articles (Garfield et al., n.d.). Among the parameters of HistCite, an article with a high global citation score (GCS) shows that this article has globally attracted scientists’ attention. However, if local citation score (LCS) of one article is high, this article has attracted scientists’ greater attention working on the same field as ours, thus, the main concentration was on LCS in this study.

## Results

### Annual publication outputs and trends

**FIGURE 2:**
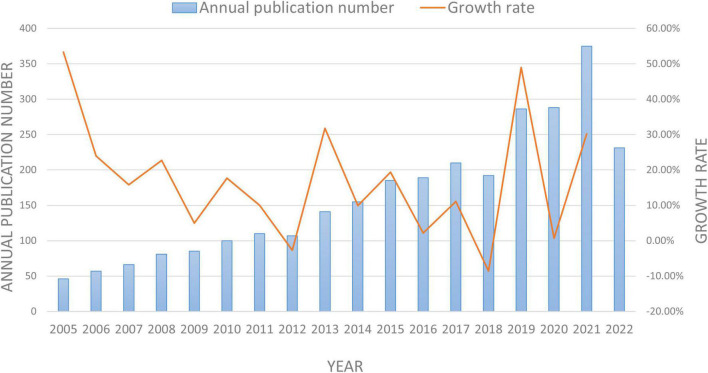
The annual number of publications and growth rate on EEG in MCI between 2005 and 2022.

Figure 2 depicts the ongoing investigation of EEG in MCI in accordance with the annual distribution of publications, with year as the abscissa and number of publications as the ordinate. With few exceptions, the annual number of articles on EEG for MCI has increased steadily from 2005 to 2022, and reached the peak in 2021. This demonstrates that this scientific field has noticeably attracted academics’ attention in recent years.

### Analysis of countries

**FIGURE 3:**
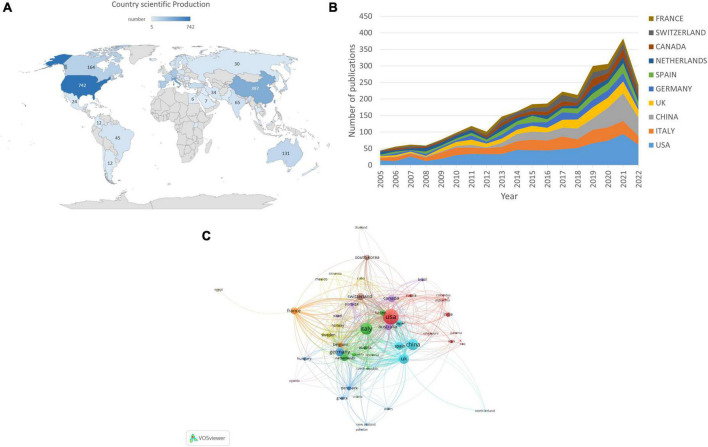
Co-authorship analysis of countries/regions in EEG research for MCI. **(A)** Geographic distribution map displaying the global distribution of EEG for MCI. **(B)** The annual number of publications from the top 10 countries/regions between 2005 and 2022. **(C)** Citation map of countries/regions generated by the VOSviewer. The size of the node denotes the number of publications, the line shows the collaboration, and the color represents the various clusters.

There have been publications on the topic published in 83 different countries/regions. The darker colors on the map indicate a high density of publications, while the lighter colors indicate a low density of publications, according to the geographical distribution of the productivity map, as shown in Figure 3A. Studies on EEG for MCI were mainly published in North America and Asia.

Electroencephalogram-related articles were published in 83 countries. Table 1 shows the top 10 countries. The USA had the highest number of EEG-related studies (*n* = 742), followed by Italy (*n* = 438), and China (*n* = 397). Regarding the number of citations, the USA had 28,206 citations, followed by Italy (13,635 citations), and Netherlands (11,711 citations). According to the centrality analysis, a node has a significant influence when its centrality is greater than 0.1. The UK (*n* = 0.25), the USA (*n* = 0.21), and France (*n* = 0.21) had a centrality higher than 0.1, indicating that these countries might play a key role in this field of study and make significant contributions. Figure 3B illustrates annual trends in the number of articles, with the USA ranking the first in terms of annual publications from 2005 to 2022. Figure 3C shows that when the number of articles was limited to >5, 52 nations were involved in the analysis of global cooperation using the VOSviewer. The top 3 countries with the highest TLS were the USA (TLS = 624), the UK (TLS = 563), and Italy (TLS = 506), according to the co-authorship visualization map.

**TABLE 1:** Top 10 countries/regions with the highest number of publications from 2005 to 2022.

Rank	Country/Region	Number of publications	Number of citations	Centrality
1	USA	742	28,206	0.21
2	Italy	438	13,635	0.08
3	China	397	4,879	0.01
4	UK	296	11,119	0.25
5	Germany	218	8,912	0.12
6	Spain	202	4,761	0.13
7	Netherlands	182	11,711	0.06
8	Canada	164	4,949	0.09
9	Switzerland	163	4,829	0.06
10	France	149	5,004	0.21

### Analysis of institutions

It was found that EEG-related articles on MCI research included contributions from 3,785 institutions, and the top 10 productive institutions are listed in Table 2. According to Table 2, among 3,785 participated institutions, IRCCS San Raffaele Pisana had the highest proportion of published articles (87, 2.30%), followed by Sapienza University of Rome (86, 2.27%), Vrije University Amsterdam (56, 1.48%), Complutense University of Madrid (54, 1.43%), and University of Foggia (52, 1.37%). The Vrije University Amsterdam, on the other hand, had the highest number of citations (6,148). However, Sapienza University of Rome had the highest centrality of 0.20.

**TABLE 2:** Top 10 prolific institutes from 2005–2022.

Rank	Institution	Number of publications	Number of citations	Centrality
1	IRCCS San Raffaele Pisana	87	2,967	0.05
2	Sapienza University of Rome	86	4,006	0.20
3	Vrije University Amsterdam	56	6,148	0.04
4	Complutense University of Madrid	54	1,481	0.03
5	University of Foggia	52	2,130	0.07
6	The University of Genoa	51	1,926	0.05
7	Università Cattolica del Sacro Cuore	44	1,486	0.05
8	University of California, San Francisco	41	3,662	0.13
9	Harvard Medical School	40	693	0.03
10	Istituto di Ricovero e Cura a Carattere Scientifico	40	1,914	0.06

**FIGURE 4:**
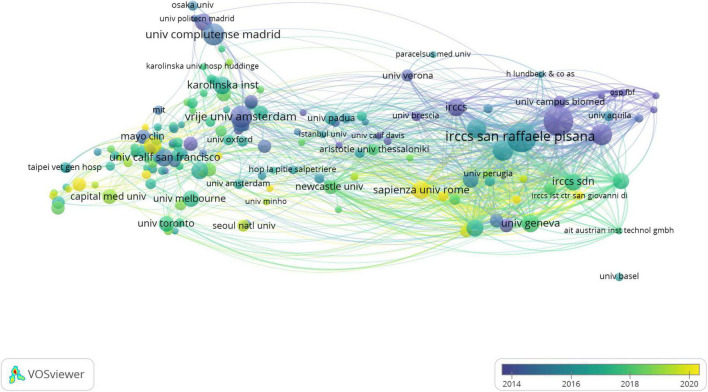
The institutions’ collaboration network visualization map generated by VOSviewer software. The size of the node denotes the number of publications, the line shows the collaboration. The color’s depth correlates to the time axis. The longer the organization has been working in this field, the deeper the color; and the shorter the organization has been involved in this field, the lighter the color.

Figure 4 shows the network visualization map created by VOSviewer to investigate institutional collaboration, including 159 nodes and 1,516 linkages. The top 3 institutions with the highest TLS were IRCCS San Raffaele Pisana (TLS = 472), Sapienza University of Rome (TLS = 386), and the University of Genoa (TLS = 327). These institutions, however, their publications were mainly belonged to before 2016. Sapienza University of Rome is the most recent organization to put the spotlight on the EEG for MCI and to collaborate in a large volume in 2020. In conclusion, the most heavily impacted institution in this field in recent years is Sapienza University of Rome.

### Analysis of journals and authors

**FIGURE 5:**
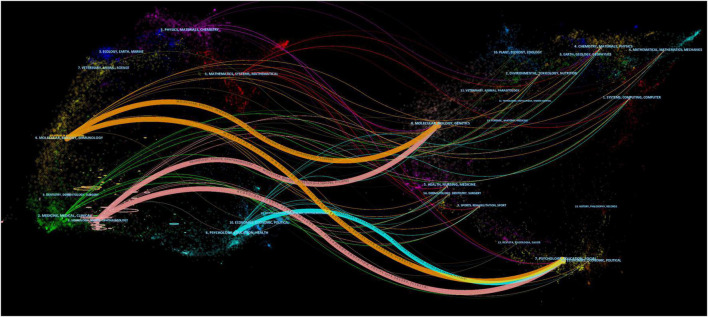
A dual-map overlay of journals related to research on EEG for MCI. The citing journals are on the left, the cited journals are on the right, and color paths indicate citation relationships. Labels were selected from journal titles to reflect relevant fields.

Electroencephalogram-related articles on MCI were published in 624 journals. As shown in Table 3, the top 10 journals published 745 articles, accounting for 25.65% of these articles. In addition, Clinical Neurophysiology had the highest number of publications (128 citations), followed by Epilepsy and Behavior, and Journal of Alzheimer’s Disease. The highest number of citations belonged to Clinical Neurophysiology (5,470 citations). Furthermore, while Epilepsia was ranked fifth, its impact factor (6.74) was significantly higher than that of the majority of the journals listed. Figure 5 is a dual-map, depicting the distribution of journals involved in EEG for MCI research, enabling scholars to comprehend the knowledge flows among various disciplines, as well as the frontiers or hotspots. On the map, the field for citing literature was on the left, and the field for cited literature was on the right. There were 5 major citation paths in this figure. The majority of the citing publications were distributed in the fields of immunology, biology, molecular, sports, ophthalmology, psychology, and education, whereas the majority of the cited articles were found in the fields of biology, molecular, genetics, psychology, education, and social sciences.

**TABLE 3:** Top 10 journals with most publications in the field of EEG for MCI.

Rank	Journal	Number of publications	Number of citations	Impact factor
1	Clinical Neurophysiology	128	5,470	4.86
2	Epilepsy and Behavior	100	1,888	3.34
3	Journal of Alzheimer’s Disease	97	2,266	4.16
4	Frontiers in Aging Neuroscience	79	1,151	5.72
5	Epilepsia	69	2,768	6.74
6	Neurobiology of Aging	66	2,943	5.13
7	International Journal of Psychophysiology	54	1,451	2.90
8	Public Library of Science	54	1,621	3.75
9	Frontiers in Neurology	53	454	4.09
10	Frontiers in Human Neuroscience	45	959	3.47

In the pertinent research, 14,067 authors were active. Table 4 shows the top 10 productive authors. Babiloni C had the highest number of publications (*n* = 84), as well as the greatest centrality (0.16). Stam CJ had the highest average number of citations per article (51.59 citations per article).

**TABLE 4:** Top 10 productive authors from 2005 to 2022.

Rank	Author	Number of publications	Number of citations	Citation per article	Centrality
1	Babiloni C	84	3,111	37.04	0.16
2	Rossini PM	82	3,266	39.83	0.02
3	Vecchio F	51	1,744	34.20	0.01
4	Del percio C	48	1,376	28.67	0.02
5	Stam CJ	37	1,909	51.59	0.03
6	Lizio R	35	1,068	30.51	0.02
7	Ferri R	34	1,043	30.68	0.01
8	Frisoni GB	33	1,473	44.64	0.04
9	Hornero R	33	825	25.00	0.00
10	Maestu F	32	820	25.63	0.07

### Analysis of references and co-cited references

The top 10 EEG-related articles for MCI research that obtained the highest LCS in the current dataset are shown in Table 5, indicating that these articles significantly contributed to MCI research. Among them, articles with the top 3 LCS were conducted by Uhlhaas and Singer (2006), Stam et al. (2007), and Stam et al. (2009), with 100, 93, and 74 citations, respectively. According to their research content, electrophysiology based on neural network was found as a hotspot research in recent years. A co-citation relationship was found when two publications were cited jointly by a third publication (Small, 1973). Table 6 summarizes the top 10 co-cited references.

**TABLE 5:** Top 10 most cited literatures.

Rank	Title	Author	Year	LCS	LCR	GCS	CR
1	Graph theoretical analysis of magnetoencephalography functional connectivity in Alzheimer’s disease	Stam CJ	2009	100	6	643	77
2	Small-world networks and functional connectivity in Alzheimer’s disease	Stam CJ	2007	93	1	927	465
3	Neural synchrony in brain disorders: Relevance for cognitive dysfunctions and pathophysiology	Peter J Uhlhaas	2006	74	3	1365	155
4	Non-linear dynamical analysis of EEG and MEG: Review of an emerging field	Stam CJ	2005	66	1	903	465
5	Epilepsy and Cognitive Impairments in Alzheimer Disease	Jorge J. Palop	2009	43	1	450	49
6	Levetiracetam suppresses neuronal network dysfunction and reverses synaptic and cognitive deficits in an Alzheimer’s disease model	Pascal E Sanchez	2012	32	3	396	70
7	Default-mode brain dysfunction in mental disorders: A systematic review	Samantha J. Broyd	2009	17	5	1,115	148
8	Amyloid-beta/Fyn-Induced Synaptic, Network, and Cognitive Impairments Depend on Tau Levels in Multiple Mouse Models of Alzheimer’s Disease	Erik D. Roberson	2011	14	1	472	73
9	Faciobrachial Dystonic Seizures Precede Lgi1 Antibody Limbic Encephalitis	Sarosh R. Irani	2011	10	0	545	32
10	Control Of Sleep and Wakefulness	Ritchie E. Brown	2012	7	2	722	1,471

LCS (Local citation score): The quantity of citations in the current dataset; LCR (Local cited reference): The quantity of articles cited in current dataset; GCS (Global citation score): The quantity of citations in the WoSCC; CR (Cite reference): The quantity of references cited by an article.

**TABLE 6:** Top 10 co-cited references involved in research on EEG for MCI.

Rank	Co-cited reference	Count	Type
1	Babiloni C, 2016, *Int J Psychophysiol*, V103, P88, DOI:10.1016/j.ijpsycho.2015.02.008	72	Review
2	Jeong JS, 2004, *Clin Neurophysiol*, V115, P1490, DOI:10.1016/j.clinph.2004.01.001	60	Review
3	Jack CR, 2018, *Alzheimers Dement*, V14, P535, DOI:10.1016/j.jalz.2018.02.018	59	Guideline
4	Albert MS, 2011, *Alzheimers Dement*, V7, P270, DOI:10.1016/j.jalz.2011.03.008	55	Guideline
5	Mckhann GM, 2011, *Alzheimers Dement*, V7, P263, DOI:10.1016/j.jalz.2011.03.005	50	Guideline
6	Babiloni C, 2006, *Clin Neurophysiol*, V117, P252, DOI:10.1016/j.clinph.2005.09.019	46	Clinical Trial
7	Koenig T, 2005, *Neurobiol Aging*, V26, P165, DOI:10.1016/j.neurobiolaging.2004.03.008	43	Clinical Trial
8	Rossini PM, 2006, *Neuroscience*, V143, P793, DOI:10.1016/j.neuroscience.2006.08.049	42	Clinical Trial
9	Pijnenburg YAL, 2004, *Clin Neurophysiol*, V115, P1332, DOI:10.1016/j.clinph.2003.12.029	39	Clinical Trial
10	Dubois B, 2014, *Lancet Neurol*, V13, P614, DOI:10.1016/S1474-4422(14)70090-0	39	Guideline

**FIGURE 6:**
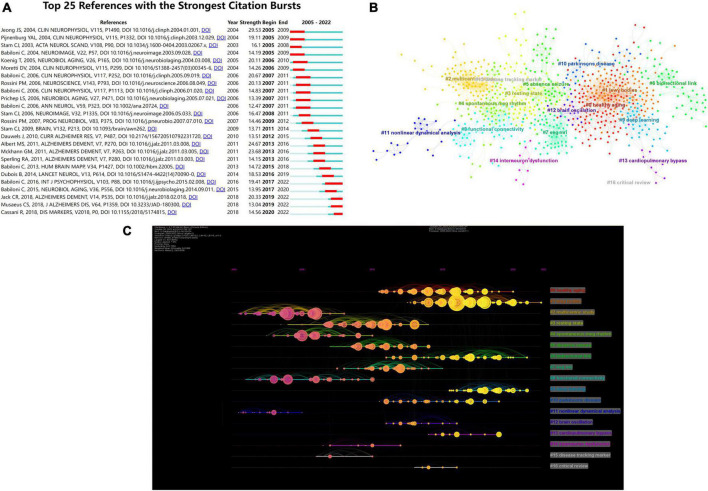
**(A)** Top 25 references with strongest citation bursts. Blue lines represent time lines, while red sections represent specific time periods of citation bursts. **(B)** Cluster analysis of co-cited references on EEG for MCI. Different colors represent different clusters. **(C)** Time evolution analysis for co-cited references. The node at the start of the horizontal axis indicates when the reference first appeared. The more yellow color means the closer to 2022, and the more pink color means the closer to 2005. The size of nodes is proportional to the number of citations of the reference. Lines between nodes indicate co-cited relationships.

Citation bursts are references that are frequently cited over time, and burst identification can emphasize the hotspots in a specific time period depending on subjects of references (She et al., 2022). In the present study, the timeline was shown by a blue line, and the intervals when bursts were explored were represented by red parts on the blue line. Figure 6A shows the top 25 articles with the greatest citation burst. The research with the highest citation burstiness (strength, 29.53) was commenced in 2005, and it specifically detailed non-linear analysis on the EEG of AD patients. According on citation bursts, the majority of these articles examined EEG from the following three perspectives. The first was to monitor the rhythm, which mainly included alpha and delta rhythms (Babiloni et al., 2004, 2006a,b,c). Other studies concentrated on synchronization or functional coupling of EEG (Stam et al., 2003; Pijnenburg et al., 2004; Koenig et al., 2005; Babiloni et al., 2016). Furthermore, some studies concentrated on power analysis of quantitative EEG, and the majority concentrated on theta power (Prichep et al., 2006; Musaeus et al., 2018). It is noteworthy that 4 references were still in the burst. These studies concentrated on theta power, synchronization, and functional coupling of resting-state EEG in diagnosing and predicting MCI and AD.

A co-citation network is a collection of references that have been simultaneously cited by one or more studies. When several texts are mentioned together repeatedly, conceptual clusters are formed (Shi et al., 2022). Clustering modularity Q and mean silhouette value were 0.7356 and 0.886, respectively, demonstrating a credible structure and clustering results. As depicted in Figure 6B, CiteSpace classified these references that were also co-cited into 17 clusters. The clustering results are presented in Table 7. As shown in Figure 6C, the individual nodes were arranged in a timeline. Co-citation links added to the year were correspondingly represented by colored curves (Chen et al., 2022).

**TABLE 7:** Specific content of cluster analysis.

Cluster	Size	Year	Label (Likelihood ratio test)
#0	123	2014	Healthy aging
#1	111	2017	Lewy bodies
#2	87	2003	Multi-centric study
#3	83	2008	Resting state
#4	63	2006	Spontaneous MEG rhythm
#5	61	2010	Absence seizure
#6	55	2016	Bidirectional link
#7	54	2011	EEG MRI
#8	54	2005	Functional connectivity
#9	45	2018	Deep learning
#10	27	2014	Parkinson’s disease
#11	23	2002	Non-linear dynamical analysis
#12	16	2013	Brain oscillation
#13	10	2016	Cardiopulmonary bypass
#14	10	2009	Interneuron dysfunction
#15	8	2007	Disease tracking marker
#16	7	2014	Critical review

### Analysis of keywords

**FIGURE 7:**
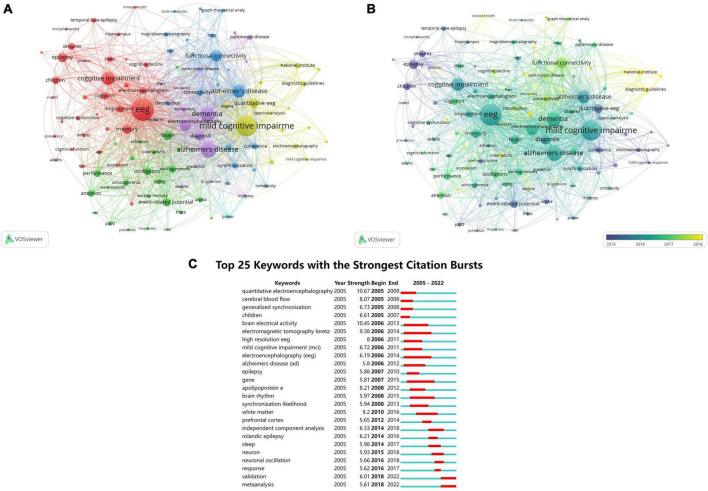
**(A)** Network visualization of keywords based on VOSviewer. The size of the node denotes the number of occurrences of the keywords, the lines indicate that they appear together in the same document, and the color represents the various clusters. **(B)** Overlay visualization of keywords based on VOSviewer. The color of the keywords corresponds to the time axis below, representing their time of appearance. **(C)** Top 25 keywords with the strong citation bursts.

Hotpots and prospective study directions in this discipline were assessed through keyword co-occurrence analysis. Through software analysis, a total of 10,149 keywords were found in 2,905 articles published from 2005 to 2022. Co-occurrence analysis produced 6 clusters, including 112 nodes, 4,644 links, and a total link strength of 33,430. Figure 7A shows the clusters according to the relationship between the weight of link characteristics under different keywords and the total link strength. These 6 clusters were cluster 1 (red, EEG), cluster 2 (green, oscillations), cluster 3 (purple, dementia), cluster 4 (yellow, mild cognitive impairment), cluster 5 (blue, functional connectivity), and cluster 6 (cyan, power). Figure 7B shows an overlay visualization map, summarizing the results of keyword co-occurrence analysis by time zone. The top 10 keywords were ranked by frequency, as listed in Table 8.

**TABLE 8:** Top 10 keywords that appeared most frequently.

Rank	Keywords	Frequency	Total link strength
1	EEG	920	7,893
2	Mild cognitive impairment	815	7,920
3	Alzheimer’s disease	546	5,047
4	Dementia	501	4,570
5	Cognitive impairment	409	3,171
6	Electroencephalography	356	3,290
7	Function connectivity	325	3,304
8	Diagnosis	259	2,201
9	Epilepsy	255	1,890
10	Memory	248	2,206

The strength of the keyword bursts is an important indicator of the study’s hotspots, as well as emerging frontiers over time (Pei et al., 2022). As illustrated in Figure 7C, the initial outbreak’s keywords were quantitative electroencephalography, cerebral blood flow, children, and universal synchronization. The strongest keyword was quantitative electroencephalography, with a score of 10.67, followed by brain electrical activity, with a score of 10.45. Notably, the citation burst time of terms, such as “validation” and “meta-analysis” (2018–2022) continued until 2022 and the signals are still present, indicating that the grade of clinical evidence for the use of EEG in MCI is currently very high.

## Discussion

### Overview of major findings

The first bibliometric analysis of articles on EEG in the MCI published from 2005 to 2022 was presented, including countries, institutions, authors, journals, historical development, and research frontiers. The number of annual publications has steadily increased in recent years, surpassing 300 for the first time in 2021. The recent development track can be divided into two stages: 2005–2016 that exhibited a slow development, and 2018–2019 and 2020–2021 that exhibited a rapid development. As a result, additional in-depth studies of the EEG for cognitive impairment are likely to be published in the coming years. Of the 10 most productive institutions, 8 were in Europe and 2 were in North America. To some extent, this demonstrates the more mature application of EEG in the developed countries. The United States and Italy had the highest number of publications and citations among more than 80 countries that have published EEG-related articles for MCI. Notably, China ranked third as the only developing country among the top 10 most productive countries. This could be related to the higher prevalence of MCI in China, which has attracted researchers’ attention. According to the statistics, the overall prevalence of MCI in China was estimated to be 15.5% by 2018, involving approximately 38.77 million patients (Jia et al., 2020). When the most cited authors were taken into account, the top 10 authors made significant contributions to EEG for MCI. The three articles published by Stam CJ were all in the top five, and their high LCS confirmed his significant contributions to the field. Stam CJ concentrated on exploring functional brain networks in cognitive impairment and used several relevant approaches for EEG analysis, including graph theoretical analysis (Stam et al., 2009), small-world network model (Stam et al., 2007), and non-linear analysis (Stam, 2005). Scholars concentrated more on the brain network mechanisms underlying cognitive impairment and other neurological disorders, and the above-mentioned EEG analysis methods capable of characterizing spatially complex brain networks were further applied and developed.

### Knowledge base

The more frequently a publication is cited, the more essential it is in a given field. As a result, the most frequently cited publication or publications with great impact factors can be regarded as a knowledge base in a specific field (Shen et al., 2022). Table 6 shows that the top 10 cited articles included 4 clinical trials, 4 guidelines, and 2 reviews.

First, 4 clinical trials concentrated on MCI diagnosis and the investigation of neurological mechanisms, particularly in association with AD. Compared with patients with subjective memory impairment, the likelihood of synchronization was significantly lower in the upper alpha and beta bands in AD, while these bands were not significantly different in the MCI group (Pijnenburg et al., 2004). This demonstrated that the diagnostic value of the synchronization likelihood in AD and MCI groups required further exploration. However, the conversion of MCI to AD could be predicted using a combined analysis of EEG rhythms by the low-resolution brain electromagnetic tomography and coherence analysis (Rossini et al., 2006). Similarly, it has been shown that cortical source analysis of the EEG rhythms by the low-resolution brain electromagnetic tomography has a predictive value to some extent in the early identification of MCI patients who may develop to AD (Babiloni et al., 2006c). Although these findings are valuable, a large number of high-quality studies are required to confirm them due to the insufficient sample size. EEG is effective not only in the diagnosis and prognosis of MCI, but also in investigating the mechanisms underlying cognitive impairment. For instance, a method of global field synchronization was used to evaluate neural networks in patients with MCI and AD, in order to examine the hypothesis of a “disconnection syndrome” (Koenig et al., 2005).

Second, all the 4 guidelines were developed by the task force released by the National Institute on Aging and the Alzheimer’s Association. Guidelines have been revised or updated mainly for MCI, as well as for core diagnostic criteria, pathophysiological properties, and biomarkers of AD. Reviews on EEG dynamics and cortical neural networks have been published separately, and they both provided a detailed summary of EEG studies on MCI and AD (Jeong, 2004; Babiloni et al., 2016).

Overall, according to the contents of the 10 highly cited references, the knowledge base in this area primarily concentrated on the diagnosis and identification of early MCI stages of AD. Several methods for EEG analysis are constantly explored and validated.

### Research hotspots and trends

By analyzing the co-citation reference timeline view, the past research hotspots and the current research trends could be found. We will focus on the clusters that are closely related to our subjects as well as those with significant impact. Figure 6C illustrates the following findings: early research concentrated on “#2 multicentric study” and “#8 functional connectivity,” interim studies concentrated on “#3 resting-state” and “#7 EEG MRI,” whereas current studies concentrated on “#1 Lewy bodies,” “#6 bidirectional link” and “#9 deep learning.”

A multicentric study approach was used in the embryonic stage of this discipline, which not only allowed for a more comprehensive reflection of the overall status of the research question, but also demonstrated the high academic impact of researchers in the field. Functional connectivity, defined as any correlation between time series of brain activity recorded across distinct brain areas, may represent interactions between dispersed brain regions (Kramer et al., 2008). Researchers have paid further attention to alterations in neural connectivity among different brain regions rather than only on focal abnormalities in specific brain regions. Numerous studies have shown that AD patients have abnormal segregated and integrated connection patterns, which are characterized by loss of small-world network properties (Stam, 2004; Stam et al., 2007). EEG-related studies further concentrated on neural networks, which could present novel perspectives into the neural mechanism in cognitive impairment.

“Resting-state” is noteworthy in this field in the medium term. A study has shown that the cortical sources of resting-state electroencephalographic alpha rhythms deteriorated across time in subjects with amnesic MCI (Babiloni et al., 2014). Compared with healthy elderly subjects, mild AD patients had a higher power of widespread delta sources and a lower power of posterior alpha sources (Babiloni et al., 2006b). Resting-state EEG rhythms can be used as a widely available marker to track amnesic MCI patients in large clinical trials.

Subsequently, EEG analytical methods were assessed. The processing of EEG signals continues to evolve dynamically, whether in the field of MCI or in other neuroscience fields. Furthermore, multiple EEG analytical methods influenced each other and developed together. As shown in Figure 6C, the “#8 functional connectivity” cluster received attention prior to 2010, indicating that investigators concentrated on cortical connectivity during this period. At this stage, the research concentration was on methods, such as connectivity analysis and graph theoretical analysis, which mainly targeted information interactions between brain regions and brain networks.

The “#7 EEG MRI” cluster suggested that multimodal neuroimaging techniques gradually developed and formed a new trend between 2010 and 2015. Multimodal neuroimaging, the main representative of EEG-fMRI, can frequently provide more complete and complementary brain information. EEG-fMRI is a technique that proposed in Ives et al. (1993) using seminal trials on patient safety and data quality, combining the high temporal resolution of EEG and the high spatial resolution of fMRI, and it has increasingly matured in recent years (Laufs et al., 2008). An EEG-fMRI study of 14 MCI subjects and 21 cognitively healthy controls indicated that in MCI, there is a clearly aberrant coupling in several networks dominated by an anticorrelation in the posterior cingulate cortex (Michels et al., 2021).

According to “#9 deep learning” cluster, with the rapid development of artificial intelligence and computer science after 2015, EEG research is shifting to a model of multidisciplinary interdisciplinary and intelligent analysis. Machine learning has emerged as the most popular and fastest growing EEG analytical method. Machine learning can extract meaningful information from noisy, multidimensional EEG signals, paving the way for automated clinical EEG analysis (Gemein et al., 2020). Deep learning is a class of machine learning, which is an algorithm developed on artificial neural networks to perform characterization learning on data by multiple processing layers. Early diagnosis and differential diagnosis of MCI have been achieved by two different deep learning architectures, including modified convolutional and convolutional autoencoder neural networks. The outcomes demonstrate a 10% increase in accuracy rate over similar research techniques, indicating that the deep learning is a promising technique for processing EEG signals (Fouladi et al., 2022).

Mild cognitive impairment detection generally involves four main steps: data preprocessing, feature extraction, feature selection, and classification (Yin et al., 2019). In recent years, many algorithms have achieved high classification accuracy rates. For example, one study utilized the piecewise aggregate approximation technique for EEG data compression, investigated the permutation entropy and auto-regressive model features, and finally utilized the extreme learning machine technique to achieve classification accuracy of 98.78% for distinguishing MCI and healthy controls (Siuly et al., 2020). Another study utilized spectral-temporal analysis to extract features from both MCI and healthy control data, derived an appropriate feature subset using a new 3-D evaluation algorithm, and achieved an accuracy of 96.94% using a support vector machine classifier (Yin et al., 2019). Recent studies have compared different machine learning and deep learning strategies for MCI detection. For instance, a deep learning framework based on the gated recurrent unit model achieved the greatest classification accuracy of 96.91% when compared to the classical machine learning strategy of early K-nearest Neighbor et al. (Alvi et al., 2022). Additionally, many methods utilizing different feature extraction and machine learning algorithms have also reached high levels of accuracy (Buscema et al., 2015; Oltu et al., 2021). Currently, most studies focus on EEG feature extraction, while signal processing methods and more advanced machine learning algorithms remain underexplored. Thus, achieving a broader range of applications requires further optimization and exploration.

Some studies have found neurophysiological links between dementia and Lewy bodies (DLB), MCI, and AD. Cortical neuronal synchronization at delta and alpha frequencies differed between ADMCI and DLBMCI patients (Babiloni et al., 2018). However, similar abnormalities have been found in AD and DLB patients during the prodromal phase of MCI. The source connectivity may reflect a common cholinergic impairment in the prodromal state of both AD and DLB because widespread interhemispheric and intrahemispheric lagged linear connectivity solutions in alpha sources were abnormally lower in this stage (Babiloni et al., 2019).

The “#6 bidirectional link” cluster refers to the existence of a pathophysiological association between AD and epilepsy. And some studies have shown links between MCI and epilepsy as well. For example, a clinical study found that amnestic mild cognitive impairment (aMCI) patients with epilepsy developed symptoms of cognitive decline 6.8 years earlier than aMCI patients without epilepsy (Vossel et al., 2013). Neurophysiological examination of a sample of 63 aMCI patients revealed that 31.75% MCI patients had epileptiform activity (Cuesta et al., 2022). The neuroelectrophysiological link between MCI and epilepsy needs more high-quality evidence to confirm, and its common pathogenesis and clinical relevance may be a hot spot for future research.

Scholars have explored the mechanisms underlying MCI and AD itself, whereas some researchers are currently searching for the intrinsic association between various disorders (e.g., epilepsy, DLB, and Parkinson’s disease) and cognitive dysfunction. In addition, the quality of a paper cannot solely be measured by citations. Some newly published articles may have lower citation counts but this does not necessarily mean they are of poorer quality. In terms of EEG analytical methods, earlier EEG spectrum analysis can provide some descriptive information; methods such as multimodal neuroimaging or graph theoretical analysis can provide us with neural network mechanisms of disease; and the latest machine learning methods can improve diagnostic accuracy. Each of these EEG analytical methods has its own advantages. Although new methods are continuously being developed and updated, some are still limited by small sample sizes and high computational costs. Future studies should fully consider the advantages of various analytical methods and explore the most suitable analysis methods for clinical applications.

### Strengths and limitations

For strengths, this is the first study to summarize the research history and development trends of EEG-related studies in the field of MCI using the bibliometric method. A variety of visualization tools were used to clearly demonstrate the results of bibliometric analysis. This study demonstrated the current *status quo*, critical issues, and future directions for EEG in MCI research. However, limitations were unavoidable. First, WoSCC database was only searched. There is a likelihood that the pertinent studies stored in other databases were overlooked. Second, because only articles published in English were retrieved, the retrieved articles might not adequately reflect all studies.

## Conclusion

In conclusion, the bibliometric analysis revealed that the application of EEG in MCI has progressed the research of a variety of diseases. The exploration of neural network mechanisms underlying MCI, and the intrinsic associations with other related disorders, by the non-stop stream of emerging EEG analytical approaches, is an important direction that remains to be addressed.

## Author contributions

ML and BL contributed to the data analysis and drafting of the manuscript. ZY visually analyzed the data. DW supervised the study and critically revised the manuscript. All authors approved the final manuscript.
